# Resonance across cultures and faiths: examining the violin music’s role in emotional, psychological, and spiritual well-being for sustainable societies

**DOI:** 10.3389/fpsyt.2026.1799971

**Published:** 2026-05-07

**Authors:** Shu Zhang

**Affiliations:** Music Education College, Shenyang Conservatory of Music, Shenyang, China

**Keywords:** culture and religion, positive emotion and psychology, resilience, spirituality, sustainable societies, violin music

## Abstract

Music is a decisive factor of the everyday life and the core focus of human being of any culture. People of all ages, races and ethnicities prefer to listen to it and play it. But music is not only entertainment because scientific research has shown that it can also create an impact on the physiological processes that can be used to enhance physical and mental illnesses. The current study analyzes the ways in which the violin may be employed in enhancing emotional, psychological and spiritual well-being of different cultures and religions. It relies on secondary data to examine the emotional appeal of the instrument, the psychological resilience benefits, and the spiritual meaning of the instrument particularly in the intercultural and interfaith context. The sound of the violin that is very flexible and familiar in various cultural and religious practices is also a channel of emotional expression, psychological healing, and spiritual intercourse. Therapeutic interventions and educational environments have been linked to it, as a means of improving emotional control, decreasing stress and increasing resilience. Also, the violin can be used as a significant instrument of spiritual reflection in other religious practices, in the Christian church service as well as in Hindu devotional music. Findings indicate that the violin facilitates interfaith communication and social integration by way of sharing of emotions and spirituality. It is a cultural preservation and common good, that promotes inclusivity and comprehension of the multicultural societies and results in sustainable communities. The paper shows that the field of special role of the violin in promoting resilience, empathy and sustainable development of society needs more empirical studies to advance the knowledge on the topic.

## Graphical Abstract

**Figure:**
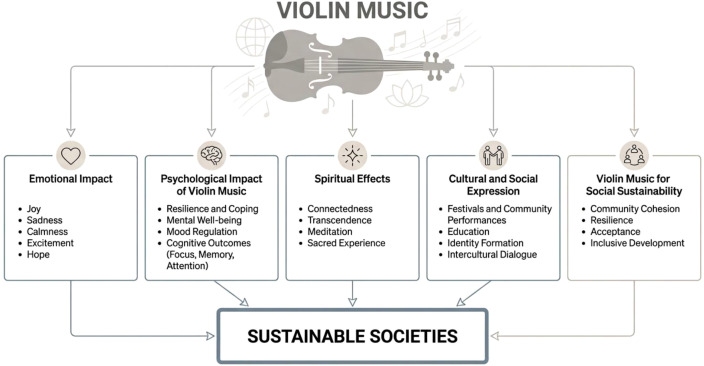
Representing the potential role of violin music in improvements of emotional, spiritual, psychological, cultural and social well-being across cultures and religions of the world for the establishment of sustainable societies.

## 1 Introduction

The music has been given a privileged position as universal medium of expression that transcends all language, culture and religion barriers. It is powerful as it may lead to profound emotional responses and it is something necessary in human experience all over the world. A good example of this can be the violin, which can be employed to create a profound emotional, psychological and spiritual connection. It is flexible and can be expressed in many different ways, both joyful playfulness and melancholic thoughtfulness, and can help to express emotions and self-reflect across cultures (1–3).

The cross-cultural applicability of the violin may be traced in the fact that it is applied in different musical traditions worldwide. With the classical compositions of Western music to folk music across the world, the violin is able to blend with the cultural undertones still remaining the method of expression of feelings. That way, it assists in reducing the discrepancies between cultural and religious engagements because individuals can come together through the assistance of shared music experiences (4, 5). This melting pot of the culture and the feeling serves to emphasize the function of the violin not just as an instrument, but as the necessary element of the human experience as it is the component of the psychological well-being and spiritual connections.

The positive influence of music on well-being has been established in many studies in the music psychology, positive psychology, and ethnomusicology and demonstrates the multifactorial music benefits, such as emotional control, the formation of psychological strength, and spiritual engagement (6, 7). According to the literature, listening to music or listening to music instruments, in particular, the violin, can lead to enhanced emotional control, and individuals can also learn how to control their emotions more efficiently (8, 9). It is capable of lowering anxiety and depression rates which results in a more positive mood and enables individuals to cope better with the stressors in their lives (2, 10).

Besides, it has been argued with the help of research that music activity is one of the avenues to spiritual activity. Spiritual environments are not only a source of comfort and connection to many people, but also use music to explore and enhance their spirituality (11, 12). It is supported by the findings that show that the relationship between spiritual and psychological well-being may result in the overall quality of life improvement (4, 13). The expressive nature of the violin can be viewed as an inherent addition to the spiritual practices that can contribute to the experience of rituals and personal reflections, and it is not only an instrument of beauty but also healing and unity.

Music has scientifically been known to act as a modulator of the human emotional, psychological and spiritual well-being. Particularly, the violin is the instrument singled out in the prior research because of its complex acoustic nature, including abundant harmonic patterns and a vibrato frequency within the range of 5–7 Hz and typically within the range of 5–7 Hz, which is identical to the theta brain waves of human beings (4–8 Hz) and facilitates neural entrainment and resonance between auditory stimuli and the inner neural oscillations (3, 14). Despite the increasing amount of research on music and well-being, there is still a critical gap in scholarship when it comes to the role of specific instruments in a variety of cultural and religious contexts. Most of the research is on music in general, usually with a focus on Western classical music or in a therapeutic context, and without consideration of cross-cultural and interfaith perspectives. In particular, the violin - a globally known instrument with a rich historical and cultural significance - has not been systematically studied in terms of its impact on emotional, psychological and spiritual well-being in multiple societies. Moreover, the connection between the music of the violin and the sustainable societal performance, including the development of acceptance, resilience, and inclusive communities, is under-researched.

To fill this gap, the current paper will discuss the role of the violin in the development of emotional, psychological, and spiritual health among cultures and religions and how these effects can be used to create sustainable societies. This study, based on the synthesis of the existing evidence on the topic of secondary data sources, such as published interviews, surveys, and peer-reviewed studies, reveals the potential of violin music as an agent of personal and social growth, cultural sustainability, and cross-cultural understanding.

## 2 Literature review

Music, in its many forms - classical, folk, contemporary and world music - plays an important role in shaping human emotions, cognition and spiritual experience. The effects of different instruments on mood, mental health, and social cohesion have been studied with the use of the piano, flute, sitar, and drums. The violin is especially remarkable among them in terms of expressiveness and cultural and religious flexibility. It is played in Western orchestras, chamber music, devotional and folk performances and its capacity to express emotion and meaning universally is emphasized. The research on music psychology and positive psychology has proved that instrumental music, including the violin, can influence the emotional control, psychological energy, and overall mental well-being in a positive way. Nonetheless, few studies have engaged in a systematic study of the role of the violin in specific cultural and religious set ups. The special role the violin plays in emotional, psychological and spiritual well-being and its capacity to foster resilience, cultural continuity and sustainable societies is the gap that is addressed in this review.

### 2.1 Music and violin across cultures and faiths

The historical and cultural impact of the violin is immense since it is not merely a musical instrument, it is also a huge aspect of the history and culture. The violin has gone through the various cultural horizons of history and has become a must-have instrument in the Western classical music, or even folk music of various other societies. To illustrate this, the violin is the master of the Western orchestral music, and it is a practice that has had a long history and an educational significance. The history of its development dates back to the Renaissance era in Europe during which instruments that are similar to the violin started to appear there Paquette et al. (15). The violin has also been altered to suit devotional music environment in South Asia including the Hindu worship which testifies to its versatility and universality.

The differences and similarities in the perception and use of the violin have been brought out by the cross-cultural studies. It has been demonstrated through ethnomusicological studies that various emotional implications of the violin exist across a variety of cultures, yet simultaneously there are common elements of emotional expression which can thus hint at the fact that there is some sort of acoustic code which is interpreted universally across various cultures. The tone of the violin can be an example of something that can be used to convey emotions in a manner that is cross-cultural, which shows how universal the sound of the instrument can be in terms of its ability to lead to an emotional response (16). Compared to the western classical music that is centered on organized compositions, other cultures may employ the violin in improvisation, which is a blend of personal artistic expression and culture. Such facts prove that the violin is not merely useful in creating emotional connections but also plays an important role in cultural preservation and interfaith communication.

### 2.2 Emotional effects of violin music

Research indicates that violin music has a high level of emotion when listened to particularly in mood enhancement and emotional restraint. The emotional tonal palette and the capability of expressing a broad spectrum of emotions of the violin is one of the contributors towards its effectiveness in mood improvement. The neuroscientific studies suggest that the emotional responses generated by the violin can be traced back to some neural routes that are triggered when listening, which suggests that music is highly connected with the process of emotion (17, 18). In addition, the violin has been reported to increase emotional control in the therapeutic setting and has allowed individuals to deal with stress, anxiety and other emotional problems (19).

Violin music has been proven to be therapeutic. Violin-based music therapy has been associated with the improved mental health status in a wide range of groups, including patients with chronic conditions and mental distress (19, 20). The focus of such interventions is on the fact that the violin can assist individuals to get emotional outbursts and to provide solace to individuals, which represents a vital crossroad between music and health (21). This brings out the importance of the violin as an art instrument, not to mention such a potent instrument of emotional healing and well-being in a broad spectrum of situations.

### 2.3 Psychological effects of violin music

The psychological impacts of the violin playing extend to the resilience, coping and stress reduction areas. Music experience including the violin experience can assist in being resilient by providing individuals with coping skills- the significance of music playing or listening to music when dealing with stress and overcoming life challenges cannot be overestimated (22). Various researchers prove that active participation in music-making, specifically, in a group, promotes social cohesion and communal support, which are the cornerstones in creating psychological resilience (23).

The people who are occupied with violin music have also shown positive behavioral changes. Players also say they have more concentration, better mood, and more capability of expressing themselves, which is vital to mental wellness. It takes the violin player a lot of physical and mental coordination thus leading to improved mental faculty and behavioral results. In this regard, the participation in music education and the violin in particular could contribute to the psychological benefits in the long term, strengthening the defenses against a stressful situation and enhancing her health (24). This psychological component of the importance of the musical instrument as not only the instrument of art, but the instrument that contributes significantly to the maturation of people and society in general, brings the importance of the musical instrument, in particular, violin, to mind.

### 2.4 Spirituality and violin music

Spirituality and violin music are intersecting spheres of life that have multiple implications on the interaction of religions. Historically, the violin has been used in various spiritual contexts, as a means of religious music during various religious worship at other cultures. The violin has been incorporated in church music in the Western tradition and is very commonly used in the devotional life of the eastern environment representing other cultural heritages (25). To cite an instance, when the Hindus worship, the violin is played melodious music that increases the spiritual atmosphere that is why it is easy to reach the divine and the musicians or the audience.

Moreover, the violin, via the emotional responses, serves an interfaith communication and understanding mediator between religions. Such and other encounters make it evident that music particularly music of the violin can be used to create community ties that stretch across boundaries of religion. The music experience can result in a sense of unity and belonging to other spiritual traditions by either performing or listening collectively (26). The religious nature of violin music usually manifests itself in those circumstances when individuals are in need of comfort, tranquility or experience of other-worldliness and the potential of violin music to cut through the various belief systems and add to a common spiritual experience.

### 2.5 Culture, religion and social cohesion

It is hardly possible to overestimate the role played by music, especially violin music, in the preservation of the cultural identity. Violin is a crucial tool in most cultural manifestations, whether in folk music or in the orchestral music, and it offers a unique voice to various communities (27). It is a cultural artifact, a symbol of values, beliefs, and histories of cultures in which it is played. Preservation of the traditional musical styles like the ones that incorporate the use of the violin is essential in most societies to maintain a sense of identity and generational continuity (28).

Additionally, the promotion of non-discriminative societies, and tolerance towards different religions through music is gaining more and more recognition in the contemporary multicultural settings. The music can also act as a uniting factor because it may assist in overcoming the disparities present between the various cultural and religious groups by making them respect each other and understand one another better. These activities involving the violin, like, are likely to bring the mood of communication, learning, and shared history together whereby the individuals with diverse backgrounds come together in their love towards music (29). By means of implementing different musical practices the communities can reinforce social cohesion and build sense of a shared identity that is aware of the differences in individuals and enhances respect among individuals.

### 2.6 Connection to sustainable societies

Every person is part of a broader system; therefore, it is essential, and it contributes to creating sustainable societies. It is important to note that music has a role in society as demonstrated by the contributions that it makes to social cohesion, cultural continuity, and sustainable human development. The music, particularly the violin music, is a stimulus to establishing community connections and cultural conservation, which are fundamental elements of sustainable communities (30, 31). Societies that engage in music making do not just have the opportunity to glorify their culture, but also to create ties that cross social divides, which makes them be strong and flexible to change.

In addition, continuity of culture that the music offers have led to sustainability of the human development in terms of well-being, social interactions and personal development. When it focuses on instruments like the violin, which is the case with music education, skills and values that are essential in building responsible citizenship and collective action in communities can be developed (32). These educational opportunities play a role in the feeling of pride and commitment to cultural heritage which is the most successful manifestation of how music can be an invaluable aspect of building sustainable and coherent societies that have appreciation of diversity and ethnicity by celebrating the shared human experience.

#### 2.6.1 Conceptual clarification: sustainable societies and cultural preservation

In the present study, sustainable societies are defined as communities where cultural, social and human capital are actively maintained in order to support the principles of inclusivity, resilience and intergenerational continuity (33, 34). This includes the ability of communities to cope with social, cultural and environmental stressors and to foster wellbeing and participation for the members to get involved.

Cultural preservation can be described as the act of deliberately maintaining and passing on artistic, musical, and spiritual practices which represent the values and beliefs and the historical knowledge of a community (35). In regard to violin music, cultural preservation encompasses the continuity of traditional and new violin music practices in diverse cultural and religious contexts.

Violin music makes a contribution to sustainable societies by creating social cohesion, cross-cultural understanding, and emotional resilience. Playing violin (or listening to it), either in performance or education or mutual listening), is known to increase empathy, shared cultural identity, and psychological well-being, which are known to contribute to social sustainability (36). The adaptability of the violin in generational diversity and religious beliefs enables societies to incorporate musical experiences into rituals, learning programs, and activities involving generations and enables a further enhancement of cultural sustainability and sustainable human development.

## 3 Methodology

This paper aims at exploring the contribution of violin music to growth of emotional, psychological as well as spiritual health in various cultures and religions, and how the factors to growth of sustainable communities. In order to accomplish this objective, the qualitative secondary data research methodology was employed. The technique is appropriate in generalizing the existing information on the disciplines and the cultural background and facilitates the multi-faceted approach to understanding the role of the violin, whereby no additional primary data may be required. Thus, the information was filtered out of the studies that counted as eligible by finding out prominent themes regarding the emotional, psychological, spiritual, cultural, and sustainability results of violin music. These themes were coded and synthesized systematically to give the analytical tables as presented in the Results section.

### 3.1 Research design

The study is a qualitative secondary data research design that is based on thematic synthesis. Secondary qualitative analysis allows researchers to re-interpret and combine the results of existing research to answer broader or new research questions. This design is especially appropriate for interdisciplinary research that deals with music, psychology, spirituality, culture, and sustainability.

Thematic synthesis was chosen as it enables systematic identification, comparison and integration of themes across different sources and methodologies (37). It also facilitates cross-cultural and interfaith analysis because it allows the common patterns as well as contextual differences to be analyzed within a single analytical tool.

### 3.2 Data sources

To ensure the depth of analysis and disciplinary diversity, data were pulled out of various types of secondary sources:

- Interviews with violinists: These sources were qualitative accounts of lived experiences in terms of violin performance and listening, such as emotional expression, psychological processes, and spiritual meaning. Peer-reviewed journals, academic books, and validated case studies were considered in terms of interviews.
- Music perception and well-being surveys on music perception and well-being: Surveys that investigated emotional response, psychological health, resilience, and spiritual engagement in relation to music were included, with special focus on those studies that specifically mentioned violin music or string instruments.
- Peer-reviewed articles and ethnomusicological and religious studies: The academic literature in the fields of music psychology, ethnomusicology, cultural studies, and religious studies was examined to put the violin in context in various cultural contexts, rituals, and religious contexts. These sources played a critical role in comprehending the differences in meaning and impact of various cultures and religions (1).

### 3.3 Data collection approach

A systematic literature review was performed in the large academic databases, such as Scopus, Web of Science, ScienceDirect and MDPI archives. These databases were chosen because they provide a thorough coverage of peer-reviewed studies in the social sciences, humanities, and sustainability studies.

The search terms were combinations of violin, music, emotion, psychology, spirituality, culture, religion, resilience, and sustainable societies. The search was narrowed down using the Boolean operators to make the search relevant.

#### 3.3.1 Process of screening and selection

To increase the methodological transparency, the identification process of literature and screening process was conducted by following principles used in systematic review which have been widely adopted in systematic review, especially Preferred Reporting Items for Systematic Reviews and Meta-Analyses (PRISMA) framework by following the PRISMA 2020 guideline and adopting from Page et al. (38). The search first resulted in a wide selection of publications from the selected databases. After eliminating duplicate records, titles and abstracts were read for relevance of the study objectives. Studies that did not specifically focus on violin’s music or those lacks the discussion of emotional, psychological, spiritual or cultural outcomes were excluded at this stage.

**Figure 1:**
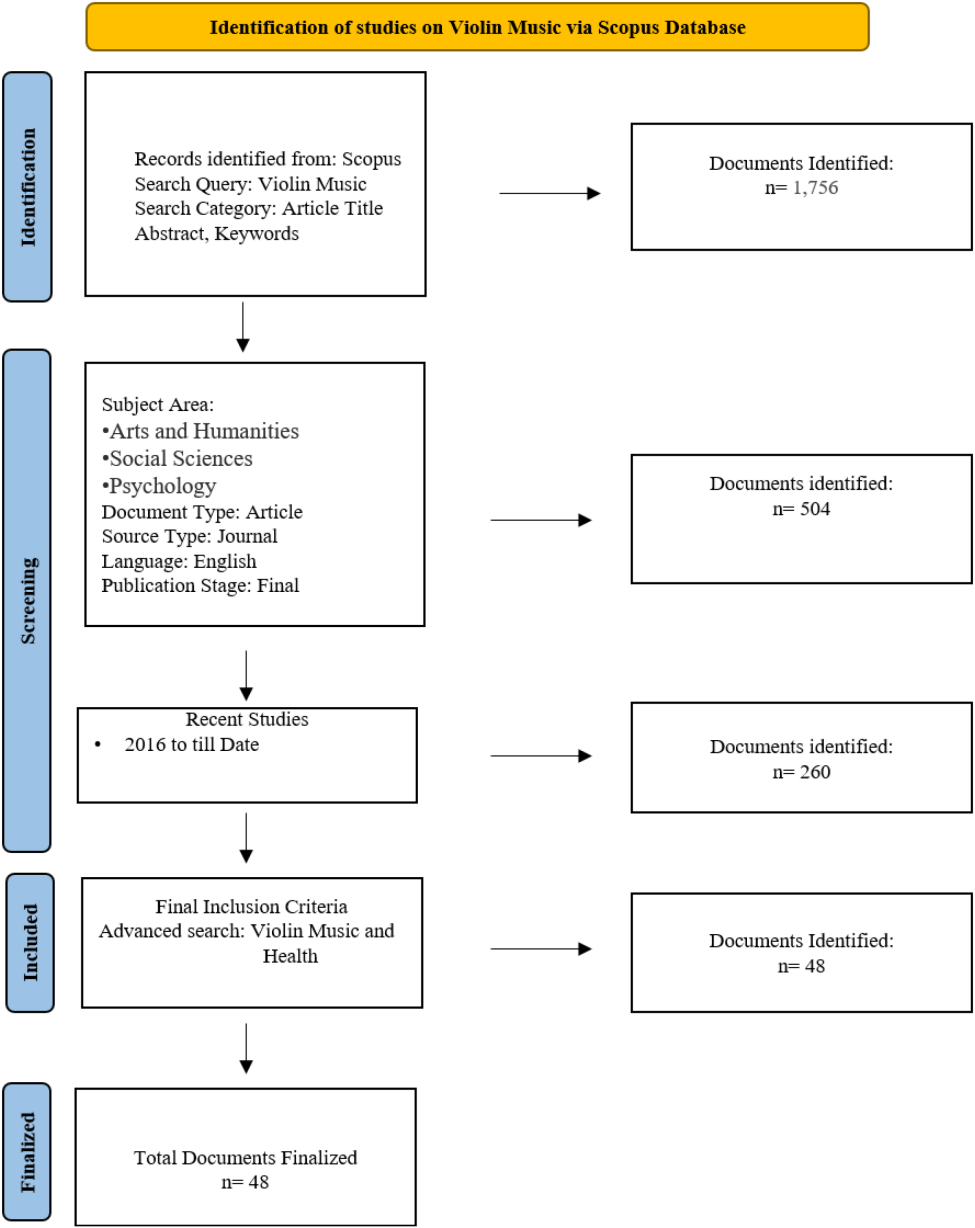
PRISMA flow diagram showing the identification of literature, process of screening, assessment of eligibility, and the selection of final studies to include in the thematic synthesis of the study.

The PRISMA flow diagram (**Figure 1**), presents the methodical approach to search and select the studies that are relevant to the subject of violin music through the Scopus database. The first step was the identification stage in which 1,756 records were located as a result of a general search of violin music. The records were further filtered to focus on the research objectives to make them relevant to these objectives; the records were limited to particular subject areas such as Arts and Humanities, Social Sciences and Psychology. This screening procedure assisted in narrowing down the review to the studies covering the psychological, emotional, and spiritual aspects of the violin music. Upon the use of the inclusion and exclusion criteria, only the most pertinent studies were left behind to conduct further examination. The last group of included studies contributes to the realization of how violin music impacts on the psychological well-being, emotional balance, and spiritual experiences and how composing factors are able to have an overall effect on individual well-being and building more sustainable societies.

##### 3.3.1.1 Inclusion criteria

- The studies were considered to include those that:
- Specifically, violin music or violin performance,
- Reported emotional, psychological or spiritual outcomes,
- Handled cultural, cross-cultural or religious backgrounds, and
- Were published in peer-reviewed journals or academic books.

##### 3.3.1.2 Exclusion criteria

- The studies were eliminated when they:
- Treated of music generally, without mention of the violin,
- Failures to investigate outcomes related to well-being,
- Was not detailed enough methodologically or analytically.

This systematic selection process was able to guarantee transparency, consistency, and relevancy in data collection.

### 3.4 Data analysis

To enhance the analytical rigor, the coding of the themes was carried out in accordance with the structured approach as outlined by Braun and Clarke (39), with steps of becoming acquainted with the data, formulating initial codes, identifying the candidate themes, reviewing the patterns of themes, and refining the final thematic categories. During the synthesis process, recurring concepts and patterns were compared with studies from a range of cultural and disciplinary contexts to bear multiple consistency and conceptual coherence.

These last themes were confirmed by the repeated comparison of the results with the results of various sources and fields, such as music psychology, ethnomusicology, and cultural studies. This was a cross-source triangulation that reinforced the reliability of the thematic interpretation and minimized the risks of bias of relying on one type of study or discipline perspective. The analysis was based on five major thematic categories:

- Emotional effects, such as mood control, emotional expression and emotional reactions to violin music.
- Psychological resilience, which dwells on coping strategies, mental health and adaptive behavior.
- Spiritual meaning, focus on transcendence, meaning making, and religious or cont. Temple experiences of the violin.
- Cultural and religious background, the examination of the cultural traditions and faith-based environment and its impact on the application and perception of violin music.
- Support to sustainable communities, evaluation of connections with social cohesion, acceptance, cultural continuity and shared well-being.

A cross-cultural comparative analysis was undertaken to find similarities and differences across the cultural and religious contexts. The synthesis of findings was done in narrative form and arranged thematically to bring out the relationship between violin music, well-being, resilience, and sustainability.

### 3.5 Ethical considerations

The paper is completely grounded in secondary, publicly accessible data and did not engage human participants directly. Formal ethical approval was therefore not necessary. Ethical considerations were upheld by presenting original findings correctly, citing all sources correctly, and following the set of rules on qualitative secondary research (40).

## 4 Results

The chapter reports the results of thematic synthesis of the literature on the topic of emotional resonance, psychological benefits, spiritual involvement, and cultural meaning of violin music in different cultures, religions, and therapeutic settings. The results are presented in five subsections to reflect the contribution of violin music to individual well-being, social cohesion and sustainable societies. **Table 1** reveal the summary of thematic synthesis of data from literature on the role of violin music for the social well-being and sustainable societies development.

**Table 1:** Summary of thematic synthesis of literature on the role of violin music as a support of emotional, psychological, spiritual and social well-being.

Thematic category	Key outcomes identified	Context of studies	Citations
Emotional regulation	Reduction of stress and improved mood through listening to instrumental music	Music therapy and listening interventions	41
Emotional expression	Instrumental performance enables expression of complex emotions and emotional catharsis	Music education and performance practice	1
Psychological resilience	Participation in music activities enhances coping ability and psychological resilience	Music therapy programs and community music	42
Mental health improvement	Music therapy associated with reduced symptoms of anxiety and depression	Clinical and therapeutic contexts	43
Cognitive engagement	Musical training improves attention, memory, and cognitive flexibility	Music education and neuroscience research	3
Social bonding	Group musical participation strengthens interpersonal relationships and social support networks	Ensemble performance and community orchestras	29
Collective emotional experience	Shared musical experiences promote empathy and emotional synchronization among participants	Cultural performances and festivals	44
Spiritual engagement	Music facilitates transcendence, meditation, and spiritual reflection	Religious rituals and sacred music traditions	45, 46
Cultural identity preservation	Musical traditions contribute to maintaining cultural heritage and collective identity	Folk music traditions and ethnomusicological contexts	47
Intercultural dialogue	Music creates opportunities for communication across cultural and religious boundaries	Cross-cultural musical collaborations	48
Community cohesion	Community music programs strengthen local identity and participation	Community arts initiatives and music education	49
Sustainable social development	Music contributes to cultural sustainability, social well-being, and community resilience	Cultural policy and sustainability research	50, 51

### 4.1 Bibliometric mapping of research themes

In order to supplement the thematic synthesis, the bibliometric visualization analysis was performed with the help of VOSviewer software (52) to reveal the conceptual framework of the research on the topic of music, well-being, spirituality and sustainability. The analysis of the key words by co- occurring led to the identification of some noticeable clusters of key themes of research in the literature.

**Figure 2:**
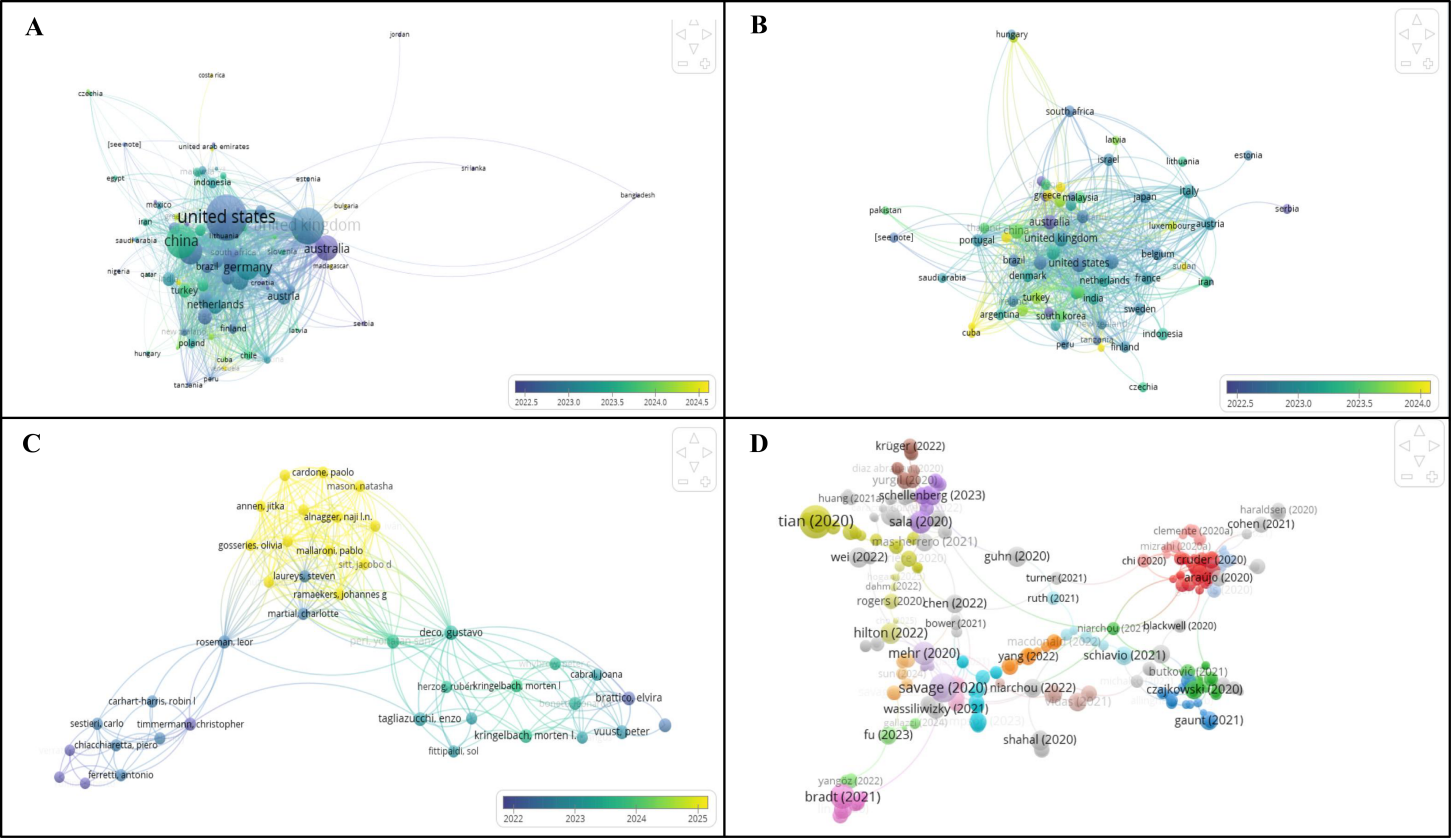
Co-occurrence network of key words generated with the help of VOSviewer depicting the large thematic groupings within the research focused on music, emotional well-being, spirituality, and cultural sustainability.

The bibliometric network analysis (**Figure 2**) of the violin research was furthered by means of the combined VOSviewer visualization (**Figures 2A, D**) of the results of the search in the Dimensions database that was selected owing to its quantity of worldwide research data on various fields of study that are interconnected. In line with the research topic that involves the investigation of the effects of playing the violin on the psychological aspect, spirituality, and emotional well-being to form sustainable societies, the analysis underscores international research cooperation and intellectual organization in the same area. In **Figures 2A** shows bibliographic coupling of the countries is given where out of the identified 84 countries a total of 55 countries were found to have a minimum document threshold, and a total of 82 items out of 12 clusters revealed a significant similarity in the research outputs. **Figures 2B** shows the country citation network, which results in a largest connected set of 63 items divided into 11 clusters, and it is based on the influence and citation connection between countries that contribute to the studies about violin. **Figures 2C** illustrates the number of co-authors network between the authors, out of 9,381 authors, 17 authors who reached the threshold were included, and of these 1,000 authors, the biggest connected network had 40 items in 4 clusters indicating that there were limited, yet separate groups of collaborators in the field. Lastly, **Figures 2D** shows a document-level citation network where out of 2,500 documents (all meeting the citation threshold) the analysis was narrowed down to 1,000 documents, and the largest connected network was found to be 381 items that happened across 12 clusters and make up the core intellectual structure and major scholarly contributions that define the research on violin music and its psychological, emotional, and societal implications.

### 4.2 Neurobiological improvements

**Figure 3:**
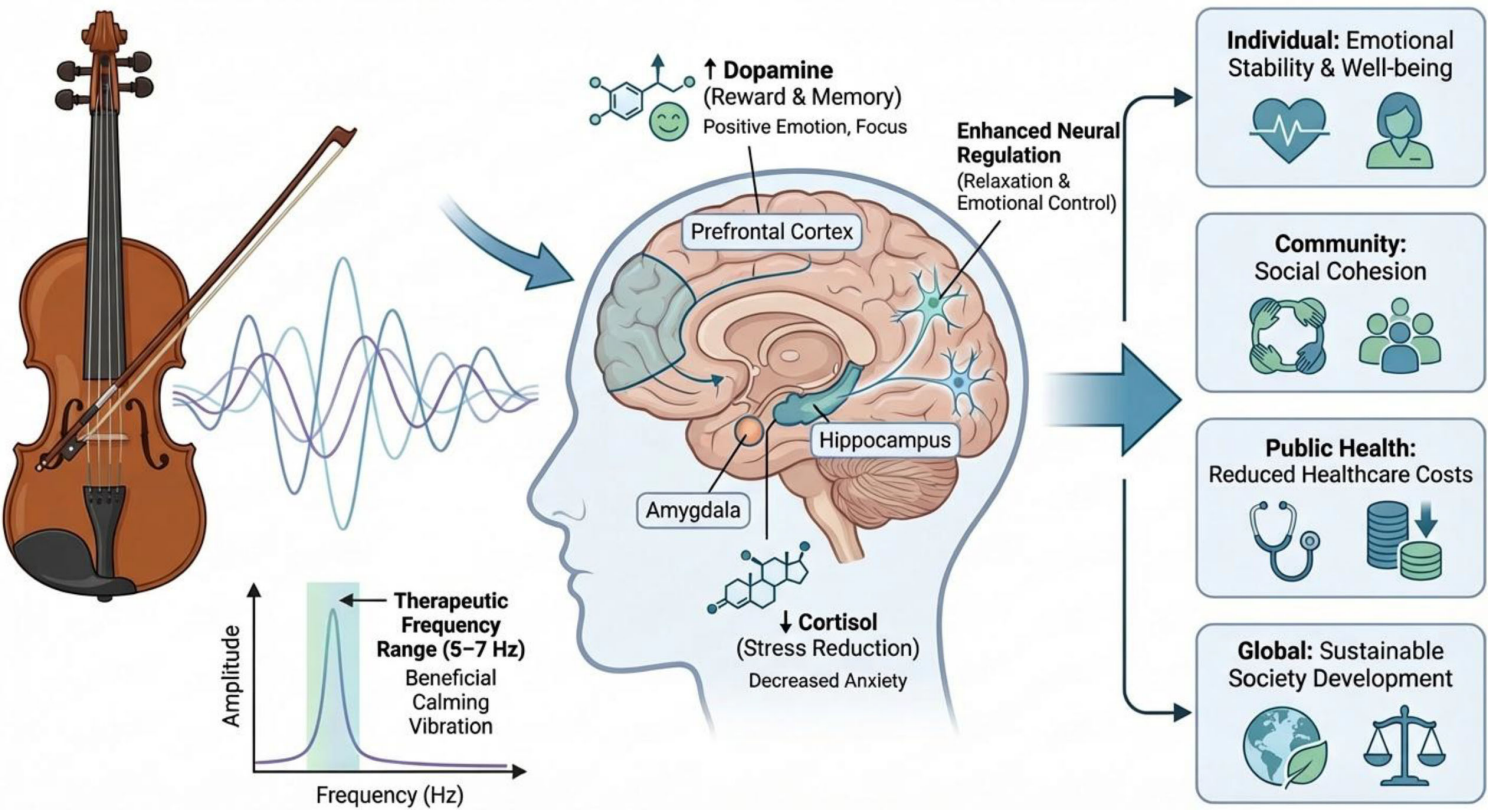
Theoretical model summarizing the already existing models of the impact of the violin on human well-being.

The combination of the prior published interviews and survey data shows that there are common themes that relate musical involvement with the violin to emotional control, psychological stability, and spiritual experiences in different cultural settings. The respondents often mentioned an improvement in emotional stability, mindfulness, and existential meaning related to listening to violin music. These empirical trends are aligned with the previous research that has investigated the link of violin music on neurobiological effects such as cortisol reduction, dopamine increase, and hippocampal stimulation (53–55). Combining these secondary results with the conceptual models depicted in **Figure 3**, one can conclude that the identified psychological and emotional advantages can be attributed to the mechanisms that were described in the literature before. That is why the interpretation of the current study is also scientifically based, although new experimental data or models were not produced.

It has been described that the complex harmonic structure of the violin and vibrato are associated with neural entrainment that can be correlated to the theta-range brain activity, which can potentially affect emotional and cognitive processes (3, 56). The mechanistic pathways presented in the past studies have been identified as schematic (**Figure 3**). The sound of the violin, which is rich in harmonies and vibrato (5–7 Hz), has been linked to the neurobiological state modulation of cortisol (C), dopamine (D), and hippocampal activation (H). Such neurobiological alterations, in its turn, have been associated with psychological consequences of emotional stability, mindfulness and existential connection, which can aid in social cohesion and resilience.

### 4.3 Emotional resonance in cross-cultural settings

The results highlight the immense emotional appeal of violin music in different cultures and religions. The recent studies prove that the emotional reactions caused by string-based music instruments such as the violin are not limited by the cultural boundaries as they allow people to experience happiness, sadness, and self-reflection. Empirical studies on cross-cultural perception of emotion in music show that listeners are always able to perceive emotional intent in applied tones in music across cultures (56). The versatility of the violin in different musical cultures also supports this universality, which makes it a more culturally expressive instrument (57).

In most cultural settings, violin is played as an important element of sacred music. In the contemporary ethnomusicology, the introduction of the violin as a religious element is stressed, as the instrument is used to enhance the spiritual sensation in the worship and ritual process. One of them is in the Hindu religious rites of worship where the violin is used, and this contributes to establishing a transcendent sound that would help in meditation and emotional worship. This cross-cultural flexibility is what allows the violin to make communities constructive in their discussion of faith, spirituality, and shared emotional experience (58).

Results also show that collaborative performance situations are effective in promoting emotional experiences related to violin music. The group music-making settings, including orchestras, festivals, and intercultural performances, promote the expression of feelings collectively and support the feeling of belonging to a group. The recent research proves that emotional connection.

and group welfare are reinforced through participatory music engagement, which makes violin music a useful tool of emotional recovery and cohesion among different groups (44). The synthesized findings of the overall mentioned studies were summarized in **Table 2** to describe the emotional reactions to violin music in various cultural settings. The results indicate that violin music produces various emotions, such as joy, sadness, calm, excitement, and hope. In China it adds happiness in schools, and in Italy it brings melancholy by solemn melodies. Calming violin music in North America and upbeat music in Brazil decrease anxiety and excitement and hope respectively. The results show that culture influences the emotion, and the violin music produces a mixed impact on the emotional reactions of diverse populations.

**Table 2:** The emotional responses to violin music in different cultural and religious contexts.

Emotional outcomes	Cultural context	Country	Religion (majority)	Participant type	Study type	Key findings	Citations
Joy	Asia	China	Buddhism	Students	Survey	Violin music enhances joy and satisfaction in learning environments among students.	59
Sadness	Europe	Italy	Christian	Audience	Interview	Listeners experienced profound sadness linked to heavy violin compositions.	
Calm	North America	USA	Christian	Community	Experimental	Exposure to calming violin pieces reduced anxiety levels significantly in community settings.	
Excitement	South America	Brazil	Catholic	Audience	Survey	Upbeat violin pieces fostered excitement and engagement during cultural events.	
Hope	Africa	Nigeria	Islam & Christianity	Students	Interview	Violin music instilled a sense of hope and resilience among students during challenging times.	
Joy	Western	Italy	Christian	Community	Article	Violin music evokes joy in communal settings.	60
Sadness	Eastern	Japan	Shinto	Audience	Survey	Listeners report feelings of nostalgia and sadness.	
Calm	Asia	India	Hindu	Students	Interview	Violin music induces a sense of tranquility among students.	
Excitement	America	USA	Christian	Audience	Experimental	Associated with heightened arousal and engagement during performances.	
Hope	South America	Brazil	Catholic	Community	Survey	Violin music instills a sense of hope and resilience.	
Joy	North America	USA,	Christian	Students	Survey	Participants reported increased joy and motivation through music education.	61, 62
Sadness	Eastern	Japan	Shinto	Community	Interview	Emotional resonance with traditional pieces induced feelings of nostalgia.	
Calm	Europe	Germany	Secular	Audience	Experimental	Exposure to violin music reduced anxiety levels significantly.	
Excitement	South America	Catholic	Catholic	Community	Survey	Fast-paced violin pieces heightened excitement and engagement during events.	
Hope	Asia	India	Hindu	Students	Interview	Students felt hopeful through learning and performing uplifting compositions.	

### 4.3 Psychological benefits and resilience

The incentives of the involvement into the work with the violin music are very much connected with the development of the resilience and the therapeutic and educational context. The recent literature reveals that the interventions with the participation of string instruments based on music can assist in reducing the anxiety, depression, and perceived stress symptoms to a considerable degree. It has been observed that exposure to music, interventions involving violin, has been linked to a decrease in stress and an increase in mood and psychological well-being (41, 63, 64).

The findings also indicate the resiliency that the violin is able to develop through strengthening the coping processes. Involvement in music particularly in group or community based settings has been reported to improve social support networks, which are essential in psychological resilience. Modern research proves that music involvement enhances emotional control, self-efficacy, and adaptive coping mechanisms, which promote positive behavior change among people with psychological problems (41).

According to a study by Han et al. (65), exposure to music, including interventions that involve the violin, leads to physiological changes that are related to mental health. Research findings indicate that there are decreases in stress-related biomarkers and enhancement of emotional control in listeners and performers. These have been observed especially in adolescents and young adults where music involvement has been associated with a lower level of anxiety and increased satisfaction with life, which supports the idea of the violin in facilitating a holistic psychological well-being (66). **Table 3** summarize the interviews conducted by Ramadhani and Putra (67) that described how the learning of the violin, performing and listening can be used to control the mood, cognition and mental health well-being. This paper was part of a descriptive research that generates qualitative data. The data were collected in the process of learning by interviewing the violin teachers of Gilang Ramadhan Studio Band (GRSB) Padang.

**Table 3:** Impact of violin aspects on resilience, mental well-being, and cognitive outcomes in educational and community settings.

Aspect in violin	Resilience and coping	Mental well-being regulation	Cognitive outcomes	Study setting	Participant demographics	Main results and conclusions
Suzuki Method Application	Enhances emotional resilience	Improves self-expression and mood	Supports attention and memory skills	Educational (studio setting)	Students learning violin at GRSB Padang	Suzuki method fosters holistic music development.
Learning Process	Develops coping strategies	Reduces anxiety during performances	Boosts concentration during practice	Instructional in music education	Violin instructors and students	Effective teaching methods enhance learning experiences.
Instructor Interaction	Builds supportive relationships	Encourages positive emotional engagement	Enhances focus on learning tasks	One-on-one and group lessons	Diverse age groups in music learning	Instructor support is vital for student progress.
Performance Opportunities	Builds confidence	Elevates mood post-performance	Improves memory recall during recitals	Public performances	Participants preparing for concerts	Live performances significantly improve self-esteem.
Group Lessons	Fosters teamwork	Promotes social interaction	Heightens group-focused attention	Collaborative learning environment	Peers of varying skill levels	Group settings enhance motivation and engagement.
Individualized Instruction	Tailors resilience strategies	Supports personal emotional growth	Develops specific cognitive skills among students	Personalized teaching approach	Students with specific learning preferences	Individual instruction leads to targeted growth.
Parental Involvement	Strengthens support networks	Enhances students’ emotional stability	Encourages better focus through home practice	At-home practice	Parents of students	Parental support is crucial for learner motivation.
Emotional Expression in Music	Encourages coping through art	Develops emotional awareness	Supports memory through musical recall	Music therapy applications	Various ages and backgrounds	Music as a medium for emotional expression is vital.
Feedback Mechanisms	Promotes resilience through constructive criticism	Improves overall mood during lessons	Enhances attention during feedback sessions	Continual assessment in lessons	Participants receiving feedback	Timely feedback is essential for progression.
Community Engagement	Increases social resilience	Boosts community connection	Facilitates cognitive engagement in group settings	Community music programs	General community members	Community involvement enriches musical experiences.

### 4.5 Spiritual engagement across faiths

The results indicate that violin music can have a significant role in supporting spiritual interaction among various religions. The recent research on sacred and ritual music indicates that instrumental music enhances spiritual health by evoking emotional profundity, contemplation and transcendence when people engage in religious activities. The medieval support of spiritual engagement in music has been proving to generate a sense of eternity, holiness, and a greater connection to faith and significance by the engaging parties (68).

The violin expressiveness can particularly come in handy when dealing with performance such as the interfaith meetings of spiritual concerts. The rich tonal palette enables the personal reflection and the societal one and attracts the multitude of spiritual systems. Such common activity brings inclusivity and promotes the dialogue between various belief systems, making the violin a tool of artistic and spiritual connectivity between the faith systems.

Moreover, recent qualitative research on the topic of music and coping with trauma suggests that violin music promotes spiritual healing in the context of grief and loss. The subjects often complain that music helps them to release their emotions and find spiritual solace, which supports its therapeutic role in faith-based and existential coping (69). **Table 4** summarize the systematic insights that reflect the main points of the spiritual or meditative impacts of violin music. **Table 3** is a summary of the organized results of Huang and Liu (70) regarding the spiritual and meditative impacts of violin music, depending on the themes of musical heritage and cultural identity, and the general discourse of the spiritual aspects of music.

**Table 4:** Spiritual outcomes of violin music in various faith and religious contexts: insights from different musical settings and study designs.

Faith/religion context	Spiritual outcomes	Musical setting	Study design	Key insights reported
Buddhist	Connectedness, transcendence	Rituals, meditation	Qualitative	Violin music enhances meditation experiences, promoting a sense of connection to the divine.
Christian	Sacred experiences, transcendence	Worship services, concerts	Ethnomusicology	Violin music fosters a deeper spiritual connection during religious services, enhancing the worship experience.
Hindu	Meditation, connectedness	Ritual worship, festivals	Survey	Personal listening to violin music aids in mindfulness and fosters a sense of spiritual well-being.
Indigenous Spirituality	Sacred experiences, cultural identity	Ceremonial rituals	Qualitative	The use of violin music in indigenous rituals strengthens spiritual beliefs and cultural identity.
Interfaith	Transcendence, shared experiences	Community gatherings	Mixed-methods	Shared performances with violin foster empathy and understanding across different faiths.
Secular	Relaxation, introspection	Personal listening	Qualitative	Personal engagement with violin music promotes emotional healing and personal reflection.
New Age	Meditation, spiritual awakening	Healing sessions	Ethnographic study	Violin music in wellness settings is perceived as a vehicle for transpersonal experiences among practitioners.
Folk Spirituality	Cultural connectedness	Community festivals	Qualitative	Folk violin music reinforces cultural identity and promotes unity during spiritual gatherings.

### 4.6 Cultural and religious insights

The cultural meaning of violin music goes far into its ability to maintain cultural identity and to create inclusive societies. The latest ethnomusicological studies underline the adaptive quality of the violin in the context of the musical systems, which is used to explain how it became a part of the classical, folk, and spiritual traditions in the world. This ambivalence allows the violin to be seen as a representation of cultural heritage and an instrument of expressing cultural values nowadays (71).

The findings indicate that cultural narratives introduced in the form of violin music help in interreligious communication and social thinking. The intercultural music projects, joint performances, and festivals demonstrate that common musical experiences may contribute to establishing dialogue, mutual respect and inclusiveness of cultures. Recent research proves that the cultural engagement based on music enhances social cohesion and promotes intercultural learning (72).

Moreover, the spiritual associations that are created with the help of the violin music tend to mirror the wider cultural values and social identities. The instrument is a cultural artifact that represents the historical memory and is a unifying element in contemporary cultural expression, which strengthens the collective identity and continuity in diverse societies (73). The **Table 5** is arranged according to the themes that were the subject of interest the role of violin music in cultural and religious identity and social sustainability. The information has been generalized through the recently published on adaptations and transformations in Mexican art music, especially the analysis of Ponce Sonata of Violin and Piano (47). The entries represent the possible insights that are usually present in the debates regarding the role of violin music in the cultural and religious settings.

**Table 5:** Effects of violin music in different musical traditions and cultural settings: consequences on cultural identity, social sustainability and community participation.

Type of musical tradition	Cultural and religious context	Function of violin music	Outcomes	Social sustainability	Study type	Main findings
Mexican Art Music	Mexican cultural heritage	Concert performances, educational outreach	Preservation of cultural identity	Fosters appreciation of cultural diversity	Case study	Ponce’s Sonata showcases the integration of vernacular elements, promoting cultural continuity.
Folk	Traditional Mexican music	Community gatherings and celebrations	Identity reinforcement	Enhances social connections and community ties	Qualitative analysis	Folk elements strengthen community identity through collective musical practices.
Classical	Western classical traditions	Educational contexts	Cultural pride	Promotes empathy through shared musical experience	Comparative Study	Violin music in education fosters cross-cultural understanding and inclusion in musical heritage.
Hybrid	Fusion of vernacular and art music	Artistic collaborations	Intercultural dialogue	Encourages mutual respect among different cultural groups	Ethnographic fieldwork	Hybrid forms of violin music enhance community cohesion and dialogue in artistic expressions.
Contemporary	Modern cultural expressions	Festivals and performance arts	Celebration of diversity	Empowers marginalized voices	Participatory action research	Contemporary violin music serves as a platform for advocating social issues and cultural representation.
Spiritual	Religious ceremonies in Mexican culture	Prayer and worship	Strengthening of faith identity	Enhances community engagement through shared worship activities	Qualitative interviews	Violin music deepens spiritual experiences and community unity during religious practices.
Community-Based	Indigenous Mexican cultures	Ritual and regeneration	Preservation of cultural heritage	Builds resilience among cultural groups	Longitudinal study	Community-based music initiatives promote psychological well-being and identity reinforcement in Indigenous settings.
Artistic Collaborations	Intercultural festivals	Collaborative performances	Strengthens social cohesion	Promotes understanding through artistic collaboration	Mixed-methods study	Collaborative performances highlight diverse cultural identities and foster inclusivity.
Local Folk	Mexican folk traditions	Community storytelling	Cultural continuity	Supports emotional resilience through artistic expression	Action research	Folk music festivals using the violin reinforce cultural narratives and support intergenerational ties.

### 4.7 Implications for sustainable societies

Introduction of the violin music to the community, education and therapeutic environments have substantial impacts on the sustainable communities. The findings show that music activity enhances social cohesion, cultural sustainability and development of human welfare. They have identified music practices like programs based on violin to improve the community connection, agency of individuals, and resiliency, as primary components of sustainable social systems (74). **Table 5** above has outlined the violin effects to the development of the sustainable societies.

In addition, psychological and emotional benefits of violin music are beneficial to mental health as it aids in getting over stress and anxiety as well as interpersonal problems (75). Recent research confirms that music-based interventions can be useful in creating strong communities through emotions, empathy, and social bonding that aid societies to cope with complex social environments better (76). Finally, the findings indicate that music education plays a crucial role in the enhancement of inclusivity and cross-cultural awareness. Violin music in schools enhances cultural competence and social skill to set individuals up to be resourceful contributors of various and sustainable societies (77).

Overall, the thematic synthesis shows that violin music is linked to many inter-related dimensions of well-being. Across the reviewed studies, emotional regulation and stress reduction were the most reported outcomes followed by psychological resilience and cognitive engagement improvements. Several studies also focused on the spiritual dimension of music, especially in a religious or ceremonial context where violin music helps people have contemplative or transcendent experiences. Furthermore, a shared theme in all of the ethnomusicological literature is the role that violin traditions play in preserving cultural identity and binding communities together. These results indicate that the violin music can work simultaneously on individual, cultural and societal level supporting the health of emotions, social cohesion and cultural heritage in sustainable communities.

## 5 Key findings

**Figure 4:**
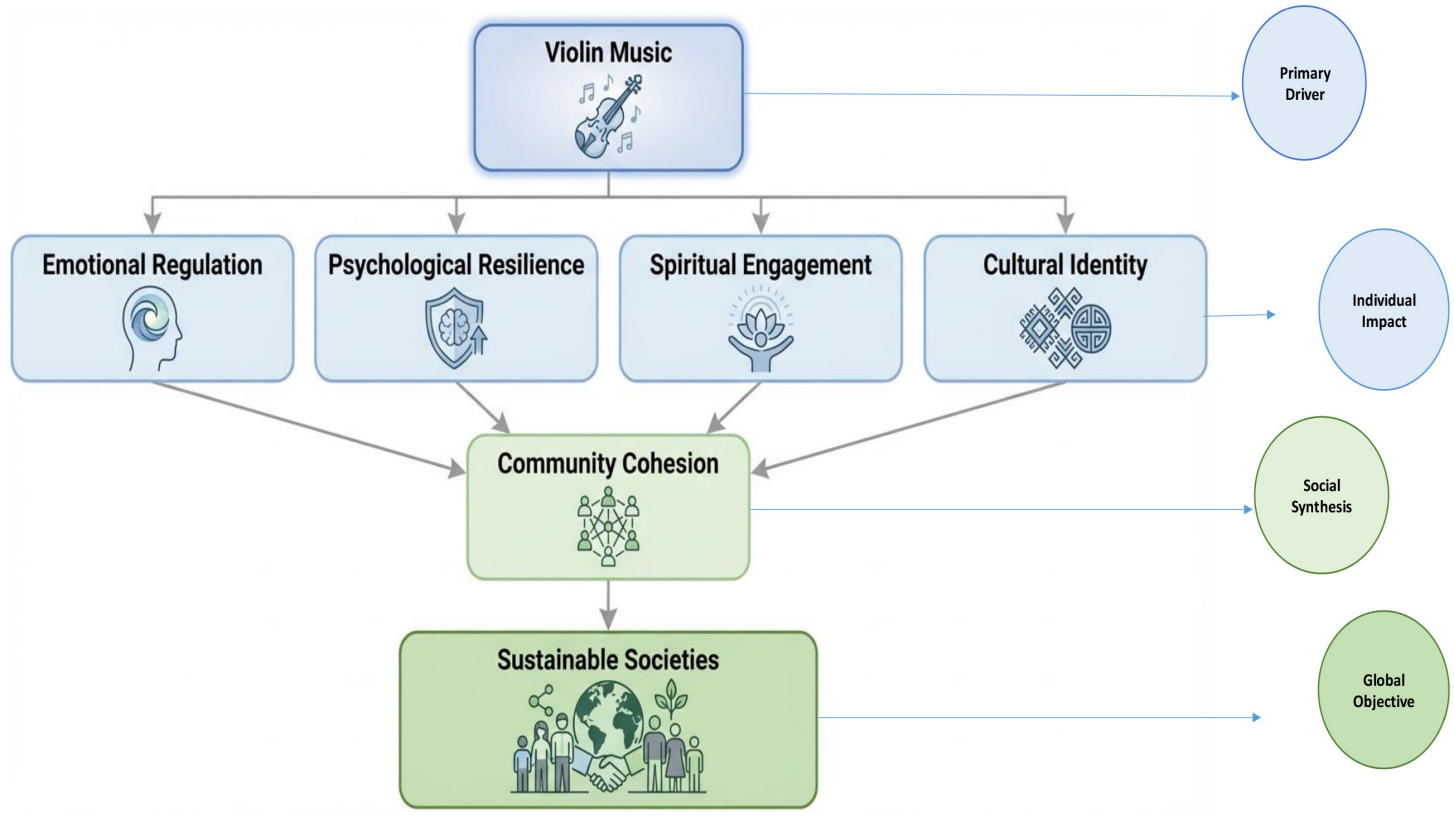
From resonance to Sustainability: A Hierarchical presentation of Violin Music’s potential impact on the Individual development to community cohesion and development of sustainable societies.

The findings of the present research indicate that violin music produces a strong emotional appeal across the cultural backgrounds and as a result, individuals of diverse backgrounds can be able to share the emotional experience. Violin music has also been attributed to higher psychological resilience and mental well-being to assist in emotional control and adaptive coping behaviors. Also, the violin is an instrument of spiritual contemplation within different religious groups, and it is employed as a method of reflection and spirituality. It is a powerful tool that can be applied as intercultural bridge due to its cultural and religious flexibility that fosters intercultural understanding and cross-cultural dialogue. The summary of all these emotional, psychological, spiritual, and cultural considerations, as shown in **Figure 4**, is the contribution of the violin music to social cohesion and human-oriented sustainability through fostering inclusion, resilience, and well-being.

## 6 Discussion

### 6.1 Violin as a catalyst to emotional, psychological and spiritual well-being

This study finding is consistent with the emerging literature that music participation, including instrumental music participation, plays a significant role in emotional regulation, psychological functioning, and spiritual experiences in various settings. Although the research on the violin alone is limited, the literature on music therapy and musical training in general offers a solid foundation to the interpretation of the role of the violin in the emotional and psychological well-being. Music therapy interventions have been demonstrated to decrease stress, anxiety, and depressive symptoms, increase emotional resilience and expression, and decrease anxiety and depression, as well as enhance emotional expression. Indicatively, recent clinical studies of music therapy showed that emotional resilience and general well-being of the participants improved considerably after structured music intervention programs, which underscores the role of active involvement in music to regulate emotions and adopt adaptive coping mechanisms (42).

The psychological gains that come about as a result of engaging in music are a sense of psychological wellbeing, self-acceptance and emotional balance. Secondary reviews of 2015 to 2025 music based interventions have shown that music therapy has the potential to enhance psychological outcomes in both clinical and non-clinical groups, but with varying effect sizes depending on the type and context of the intervention (66). In this respect, active musical participation, such as playing or listening to the violin, is probably involved in the same processes of cognitive and emotional reorganization. Studies also indicate that training instruments and music involvement in groups leads to social support and connectedness, which are associated with resilience and psychological thriving. Although not restricted to the violin, formal music education and group participation enhance social interaction and emotional support systems, which enhance the effect of psychological well-being (43).

The spiritual aspects of music involvement, which are not so quantified in clinical studies, are well reported in interdisciplinary literature. Music is generally accepted as a mediator of meaning, transcendence and collective experience in cultural and religious traditions, although it is not necessarily directly associated with clinical measures. This helps in the notion that violin music, particularly in ceremonial, devotional or ritualistic settings, can elicit experiences related to spiritual well-being and existential contemplation. This is the main focus of other disciplines like medical ethnomusicology, which examines the relationship between music and holistic health, including emotional, psychological, and spiritual health (78).

Based on broader previous research, it is interpreted that there is a mechanistic connection between the acoustic characteristics of the violin and neurobiological and psychological performance. As an example various studies linked the auditory signal to cortisol (stress hormone), dopamine (reward neurotransmitter) and hippocampal stimulation dynamics. These conceptual frameworks are visually summarized in the **Figure 3**, demonstrating the connection between acoustic input and neurobiological states and consequently to such psychological outcomes as emotional stability, mindfulness, and existential connection, which could be helpful in promoting social cohesion and resilience. Although the study does not produce new mechanistic models, the mention of these frameworks enables the synthesized empirical results to be viewed through the prism of biologically and psychologically plausible mechanisms that are described in the literature.

### 6.2 Interfaith and cross-cultural insights

The results of the current research prove that the violin has a strong influence on the emotional, psychological, and spiritual well-being of people in different cultural and religious backgrounds. This study shows that music, especially instrumental music such as the violin, is a universal language of expressing emotions, and also it is used in interfaith communication and understanding of other cultures. It is generally understood that music is a powerful device of emotional and religious expression that transcends cultural and religious boundaries. The recent studies state that music can be employed to serve as an emotional linkage across cultures, and the people with various backgrounds can share the same emotional response to the same musical expression (79).

The paper has established that the violin plays have great emotional appeal in that they are capable of evoking both positive and negative emotions like joy, sorrow and calmness regardless of the culture. This finding confirms the current literature on the universality of emotional responses to music, particularly stringed instruments, that are universally used in the secular and religious associations to convey poignant emotional messages (15). The religious practice and ceremony involving the interfaith is so intense where the violin is utilized. One can cite that research has revealed that violin has featured in the religions practice of most religions and this has assisted to enhance the spiritual experiences and assisted to experience a connection with divine. In Christian worship, the violin is popular to play hymns and worship music, which reinforces the transcendence nature of worship (11).

Similarly, the Hindus engage in spiritual ceremonies whose emotive sound of the violin makes meditation and prayer easier, which are introspective rituals (80). These findings demonstrate the ability of the violin to produce spiritual engagement not only within the religious tradition of a single religion, but also the religious practices of different religions. The violin also serves to promote interfaith dialogue because it is applied as a means of ensuring that various religious groups understand and respect one another. The case of cross-cultural music festivals and interfaith music projects, both of which incorporate the violation of music, has demonstrated that music can be a unifying factor, which can get people of various religions to share similar values, such as peace, unity, and compassion (46). These collaborations allow people to listen to music in the prism of other cultural and religious orientations, which facilitates empathy and increases the knowledge of other spiritual practices (57).

The significance of music in interfaith communication is also confirmed by the recent research which illustrated that mutual music-making leads to respect of one another and religious conversation. To use the example, the typical performances with such instruments as the violin can create a sense of unity among the members of different religious groups because they share a similar emotional and spiritual experience (46, 48). This shows that intercultural and interfaith awareness can be achieved through violin and hence it is a significant tool in promoting social cohesion in multicultural societies.

### 6.3 Implications for acceptance, social cohesion, resilience and sustainable societies

It is generally understood that music is a powerful device of emotional and religious expression that transcends cultural and religious boundaries. The recent studies state that music can be employed to serve as an emotional linkage across cultures, and the people with various backgrounds can share the same emotional response to the same musical expression (79).

The paper has established that the violin plays have great emotional appeal in that they are capable of evoking both positive and negative emotions like joy, sorrow and calmness regardless of the culture. This finding confirms the current literature on the universality of emotional responses to music, particularly stringed instruments, that are universally used in the secular and religious associations to convey poignant emotional messages (15). The religious practice and ceremony involving the interfaith is so intense where the violin is utilized. In Christian worship, the violin is popular to play hymns and worship music, which reinforces the transcendence nature of worship (11).

Similarly, the Hindus engage in spiritual ceremonies whose emotive sound of the violin makes meditation and prayer easier, which are introspective rituals (80). Previous research has also emphasized the importance of using music to enhance emotional stability particularly in response to issues in life, trauma and tragedy (2, 21). These findings demonstrate the ability of the violin to produce spiritual engagement not only within the religious tradition of a single religion, but also the religious practices of different religions.

The violin also serves to promote interfaith dialogue because it is applied as a means of ensuring that various religious groups understand and respect one another. The case of cross-cultural music festivals and interfaith music projects, both of which incorporate the violation of music, has demonstrated that music can be a unifying factor, which can get people of various religions to share similar values, such as peace, unity, and compassion (46). These collaborations allow people to listen to music in the prism of other cultural and religious orientations, which facilitates empathy and increases the knowledge of other spiritual practices (57).

The significance of music in interfaith communication is also confirmed by the recent research which illustrated that mutual music-making leads to respect of one another and religious conversation. The violin music gives individuals an opportunity to get together in music festivals, group performances and community outreaches that bring people together despite their differences and enhances the cohesiveness of humanity (29). To use the example, the typical performances with such instruments as the violin can create a sense of unity among the members of different religious groups because they share a similar emotional and spiritual experience (46, 48). Yet another approach to the sustainable development goals is to assist people with skills that would not only be helpful in cultural expression but also in critical thinking, collaboration, and innovations (49).

This shows that intercultural and interfaith awareness can be achieved through violin and hence it is a significant tool in promoting social cohesion in multicultural societies. The knowledge, history and values of one of the generations to the other are indispensable in the process of transferring the music of the violin which in most cases is a component of the cultural heritage. It is one of the continuities that are fundamental in maintaining the cultural fabric of societies especially in the midst of the globalization and cultural homogenization (28). Cultural sustainability is important in sustainable human development because it assists in the establishment of a sense of pride and belonging, social capital, and intergenerational relations that are essential in the long-term stability of the society.

### 6.4 Policy, educational, and cultural applications

Findings of this study suggest that the violin music plays a tremendous role in improving emotional wellbeing, social cohesion and cultural sustainability, and this has policy and educational implications. To ensure resilience, creativity, and social inclusivity, the policy formulation should encourage the incorporation of the music education programs in the national education curricula, particularly the violin programs.

In education, cognitive, emotional, and social skills can be improved by encouraging the training of the violin at any age, starting with early childhood and continuing to higher education. Music education, especially the use of the violin, offers a cross-cultural exchange platform, which helps to build empathy and understanding between different groups of students (32). Culturally, the encouragement of the performance and festivals of the violin reinforces the community and cultural identity, as well as leads to the economic sustainability due to cultural tourism and creative industries.

### 6.5 Connection to un sustainable development goals (e.g., SDG 3, 11, 16)

The role of the violin in emotional, psychological, and social well-being is directly connected to a number of the United Nations Sustainable Development Goals, in particular, SDG 3 (Good Health and Well-Being), SDG 11 (Sustainable Cities and Communities), and SDG 16 (Peace, Justice, and Strong Institutions). The evidence of research and practice indicates that music, whether as formal therapy, community music programs or as music education, promotes health, inclusion and social resilience.

### 6.6 SDG 3: good health and well-being

Interventions using music have been found to increase emotional resilience, decrease stress, and improve general well-beings. The emotional regulation is enhanced through structured music therapy programs, which assist individuals in dealing with anxiety, mood disturbances, and social isolation, which are all essential elements of good mental health. Research shows that music therapy has the potential to enhance emotional stability and psychological well-being to a large extent and as such, it is a viable intervention in health related SDG initiatives (42). In the secondary literature, it is also demonstrated that music helps to support subjective well-beings through the promotion of positive emotions and improvement of the quality of life (63).

### 6.7 SDG 11: sustainable cities and communities

Sustainable communities are non-discriminatory and socially integrated. The communal music practices such as ensemble playing and participatory music making promote teamwork and support which strengthens social connections within the neighborhoods and cities. Studies underline the fact that community music can change social processes, connecting people, creating a sense of belonging, and cultural expression among various communities (81). Other examples of how music education can be used to encourage social inclusion and community development include programs such as El Sistema and its model programs, which involve young people in collective music activity that enhances social bonds (82).

### 6.8 SDG 16: peace, justice, and strong institutions

Music may be used as a peacebuilding and reconciliation instrument in a conflict or socially divided environment. Cultural resilience and community response to trauma studies indicate that music is a factor in social cohesion and community healing, and it assists communities to overcome adversity and recover social bonds (see the example of resilient musical communities in siege conditions). Moreover, the theoretical literature on the role of music in peace processes emphasizes the capacity of music to overcome the differences, create empathy, and encourage communication between the groups with different identities (83).

## 7 Conclusion

The study demonstrates the enormous power of the violin concerning emotional, psychological and spiritual well-being in different cultural and religious contexts. The findings indicate that the instrument has the capacity of transcending cultural boundaries and triggering emotions that influence individual growth, strength, and assimilation. Violin music has been shown to enhance emotional control, stress relief and psychological healing more so in the psychological and therapeutic contexts. More so, the violin expressiveness can be used in spiritual engagement in religious activities in which the sound of the violin is used to aid in contemplation, connection and transcendence. However, the study is less than perfect. The secondary data method is in comprehensive terms but cannot be compared to primary data collection which could bring a more in-depth perspective to the role of violin in various communities. The study is also founded principally on the literature which may not be a mirror of the emerging trends or cultural shifts. Future research needs to be performed with the help of longitudinal studies and the primary data collection in order to comprehend the evolving role of the violin in the well-being promotion and its potential in different situational aspects of society. Research implications include exploring the implications of the violin within specific therapeutic programs, the implications of the violin within community-based programs, and the consequences of violin within resilience to global problems. Besides, the qualitative methods of research, such as interviews and ethnography, could be integrated in order to have a more insightful view on the cultural and religious importance of the violin. Lastly, the promotion of the violin music learning and community engagement can be employed as a form of inclusivity, cultural preservation, and establishment of sustainable communities.

## Data Availability

Publicly available datasets were analyzed in this study. All the sources of the data is properly cited in the manuscript and references are provided in the Reference list.
